# Carbon Membranes Derived from Natural Polymer Precursors: Fundamentals, Developments, and Perspectives for Pervaporation Desalination

**DOI:** 10.3390/membranes15120354

**Published:** 2025-11-25

**Authors:** Yue Yuan, Fang Wang, Yin Yu, Zhikai Qin, Hongbo Xi, Changyong Wu

**Affiliations:** 1 Research Center of Environmental Pollution Control Technology, Chinese Research Academy of Environmental Sciences, Beijing 100012, China; yuan.yue@craes.org.cn (Y.Y.); wangfang235@mails.ucas.ac.cn (F.W.); yu.yin@craes.org.cn (Y.Y.); qinzhikai@hhu.edu.cn (Z.Q.); 2 College of Hydrology and Water Resources, Hohai University, Nanjing 210098, China

**Keywords:** carbon membrane, natural polymer precursor, pervaporation desalination, biomass-derived carbon, sustainability, structural stability

## Abstract

Carbon membranes have emerged as a promising class of inorganic membranes for desalination due to their tunable pore structures, superior chemical and thermal stability, and molecular-sieving properties. In pursuit of sustainability, recent research has shifted focus towards replacing petrochemical-based precursors with renewable natural polymers. This review provides a comprehensive examination of the fundamentals, developments, and prospects of carbon membranes derived from natural polymer precursors—such as cellulose, chitosan, lignin, starch, and sugars—specifically for pervaporation desalination. It begins by summarizing the fundamentals of membrane separation and the mechanisms of carbon membrane formation, emphasizing the critical relationships between precursor structure, carbonization conditions, and the resulting membrane performance. The core of the review is dedicated to a detailed analysis of various natural polymer precursors, discussing their unique chemistries, carbonization behaviors, and the characteristics of the derived carbon membranes. Particular attention is given to their application in pervaporation desalination, where they demonstrate competitive water flux and high salt rejection (>99%) under moderate operating conditions, highlighting their potential for treating hypersaline brines. Finally, the challenges of large-scale fabrication, structural durability, and data-driven optimization are discussed, along with future directions toward scalable and sustainable membrane technologies.

## 1. Introduction

The availability of freshwater resources for human consumption, industrial use, agriculture, and emerging energy technologies such as hydrogen production via electrolysis has become an increasingly critical global challenge [1,2]. Climate change, rapid industrialization, and population growth have intensified the demand for freshwater while degrading natural water quality. As a result, desalination has emerged as a vital and rapidly expanding technology for securing water supply. Traditional thermal desalination methods, such as multistage flash (MSF) and multiple-effect distillation (MED), are effective but highly energy intensive. In contrast, membrane-based desalination technologies, including reverse osmosis (RO), membrane distillation (MD), and pervaporation (PV), have become dominant due to their lower energy requirements [3]. Among these, PV is chosen as the focus of this review for carbon membranes due to its unique advantages in treating hypersaline brines, which are challenging for RO, and its lower susceptibility to fouling compared to porous membrane processes like MD.

Despite the significant progress achieved in the past two decades, polymeric membranes remain the mainstream materials in commercial desalination systems. However, these membranes often suffer from limitations such as low thermal and chemical resistance, membrane fouling, and structural instability under harsh operating conditions [4]. These constraints hinder their long-term operation and limit their applicability in treating hypersaline or chemically aggressive streams. In response, inorganic membranes, such as titania, silica, zeolite, and carbon membranes, have attracted growing interest for their superior mechanical and chemical stability, tunable pore structures, and exceptional selectivity [5,6,7,8]. Among them, carbon membranes stand out as a promising class of inorganic membranes that combine high temperature tolerance with molecular-sieving capability.

**Figure 1:**
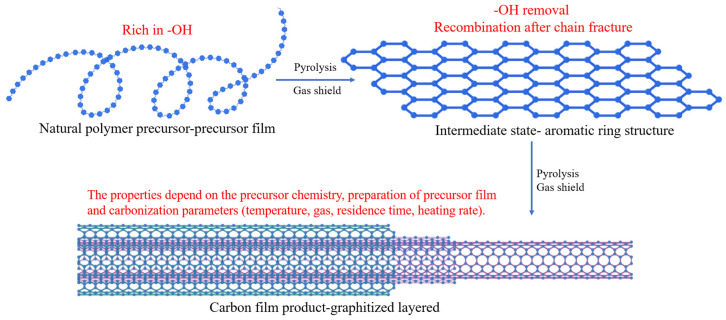
Carbon membrane formation schematic.

Carbon membranes are typically fabricated by controlled pyrolysis of organic precursors, producing a rigid carbon matrix with micropores that can selectively allow the passage of specific molecules or vapors. The fundamental chemical transformation involved in this process, from polymer precursor to a porous carbon structure, is schematically illustrated in Figure 1. Owing to their tunable pore structures, high stability, and resistance to organic solvents, carbon membranes have been extensively studied for gas separation, vapor permeation, and, more recently, desalination and water purification [8,9,10]. The performance of carbon membranes is intimately tied to the chemical composition and morphology of their precursors, as well as to pyrolysis parameters such as heating rate, atmosphere, and final temperature [11]. Commonly used precursors include polyimide (PI) [12], polyacrylonitrile (PAN) [13], phenolic resin [14], and polyfurfuryl alcohol (PFA) [15], etc. However, the heavy reliance on synthetic polymers derived from petroleum feedstocks poses sustainability and cost concerns.

In recent years, driven by the principles of green chemistry and sustainable materials development, researchers have begun exploring renewable natural polymers as alternative precursors for carbon membrane fabrication. Natural biopolymers such as cellulose, chitosan, lignin, and starch offer advantages including abundance, biodegradability, and low cost. The conversion of these renewable macromolecules into functional carbon structures aligns with global trends toward circular economy and low-carbon manufacturing [16]. Moreover, the diverse molecular structures and abundant functional groups of natural polymers can facilitate tailored pore development and heteroatom doping during carbonization, enhancing adsorption and transport properties.

The past few years have witnessed accelerated progress in this area. For example, cellulose-derived carbon membranes have demonstrated competitive performance in gas and vapor separation [17], while lignin and chitosan have shown potential for fabricating nitrogen-doped carbon frameworks with enhanced hydrophilicity and stability [18,19]. Similarly, bio-derived carbons from sugars and starch have been explored for producing carbon materials and membranes with hierarchical porosity [20,21,22,23]. These developments collectively mark a significant step toward sustainable membrane materials and open new pathways for environmentally friendly desalination technologies.

Despite significant progress in carbon membrane technology, existing reviews primarily focus on synthetic polymers such as polyimide and carbon nanomaterials as precursors, with an emphasis on their applications in pressure-driven water treatment processes like gas separation, batteries, and reverse osmosis/membrane distillation. These studies mainly address process optimization, doping modifications, and structure–performance relationships [24,25,26,27,28,29]. However, two major gaps remain: first, the potential of natural polymers as sustainable precursors has been overlooked; second, there is a lack of specialized analysis on pervaporation desalination.

Therefore, this review aims to systematically evaluate the application of natural polymer-derived carbon membranes in high-salinity pervaporation desalination, establishing intrinsic links between precursor chemistry and desalination performance in the context of sustainability. The article comprehensively outlines the fundamental structure–property relationships and fabrication methods of carbon membranes prepared from natural polymer precursors, as well as their applications in desalination. It also highlights recent advances in the design and performance of bio-based carbon membranes, and discusses future directions for achieving high-performance, sustainable, and scalable membrane systems.

## 2. Membrane Separation and Carbon Membrane Fundamentals

### 2.1. Membrane Separation Technologies

Membrane separation is a physicochemical process that relies on selective mass transport through a semipermeable barrier. The driving force may be pressure difference (∆P), concentration difference (∆C), temperature difference (∆T), and electrical potential difference (∆E) [30], as summarized in Table 1, depending on the separation mode. Common technologies include microfiltration (MF) [31], ultrafiltration (UF) [32], nanofiltration (NF) [33], reverse osmosis (RO) [34], pervaporation (PV) [35], and membrane distillation (MD) [36]. The selectivity and permeability of each process are governed by the membrane’s pore structure, surface chemistry, and the nature of the driving force [37].

Reverse osmosis (RO) membranes dominate large-scale desalination owing to their ability to reject nearly all dissolved salts. However, RO systems operate at high pressure (40~80 bar for seawater), suffer from fouling, and are limited by the intrinsic permeability-selectivity tradeoff of polymeric materials. Nanofiltration (NF) offers lower energy consumption and partial ion rejection, making it suitable for brackish water and wastewater reuse. Membrane distillation (MD) and pervaporation (PV) are thermally driven processes capable of treating hypersaline or organic-laden streams that are beyond the osmotic limits of RO. In PV desalination, water evaporates through a dense hydrophilic layer and condenses on the permeate side, enabling near-complete salt rejection (>99.9%) while using moderate temperatures (50~80 °C). Recent research highlight steady progress in hybrid PV membranes incorporating inorganic or carbon-based fillers, which enhance hydrophilicity, structural stability, and anti-fouling properties. For example, Xie et al. [38] and Chaudhri et al. [39] reported that incorporating SiO_2_ into pervaporation desalination membranes can enhance membrane hydrophilicity, thereby increasing water flux while maintaining a salt rejection rate of over 99%.

### 2.2. Membrane Classification

**Figure 2:**
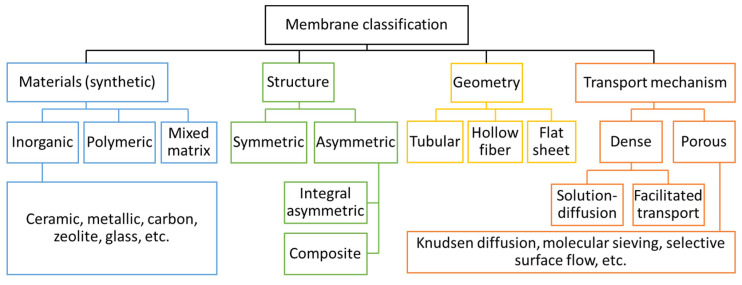
Different classifications of membranes.

Membranes can be categorized according to many different classification schemes such as materials, structure, geometry, and transport mechanism, as shown in Figure 2. In the materials characterisation, membranes can be polymeric, inorganic, or hybrid. Each different material has its own advantages and disadvantages.

#### 2.2.1. Polymeric Membranes

Polymeric membranes occupy the majority of membrane market. The most widely used materials for polymeric membranes include cellulose acetate (CA), polysulfone (PSU), polyethersulfone (PES), polypropylene (PP), polyacrylonitrile (PAN), polyvinyl alcohol (PVA), polyimides (PI), polytetrafluoroethylene (PTFE), and polyvinylidene fluoride (PVDF) [40]. The key features of some of the materials are in Table 1. Phase inversion, interfacial polymerization, stretching, track-etching, and electrospinning are some common methods to fabricate polymeric membranes [41]. Although polymeric membranes have been successfully utilized in industries due to their low cost, flexibility and high efficiency, problems like fouling, swelling, chemical and thermal susceptibility still exist and limit their application [42]. Therefore, to tackle these problems, block copolymer membranes and surface modification methods like surface grafting and surface segregation have been developed by researchers [43,44].

**Table 1:** Common polymeric membrane materials [40,41,45,46,47].

Materials	Technology Maturity	Applications	Advantages	Disadvantages
Cellulose acetate (CA)	Commercial	RO NF UF MF	Low cost Flexibility in fabrication Hydrophilicity	Poor chemical and thermal stability
Polysulfone (PSU), polyethersulfone (PES)	Under development	NF UF MF Gas separation	High mechanical strength and durability Good chemical resistance and chlorine resistance Wide pH tolerance High thermal stability High permeability	Hydrophobicity Low operating pressure limits Low fouling resistance
Polypropylene (PP)	Commercial	UF MF MD	High resistance to organic solvents Good mechanical strength	Low fouling resistance Not oxidant tolerant
Polyacrylonitrile (PAN)	Commercial	UF MF Pervaporation	Oxidant tolerant Narrow pore size distribution	Poor thermal stability Low fouling resistance
Polyvinyl alcohol (PVA)	Under development	Pervaporation	Hydrophilicity Good acid resistance High fouling resistance	Poor mechanical stability Low pressure resistance
Polyimides (PI)	Under development	Gas separation	High thermal stability High mechanical properties High chemical tolerance	Polymer chain rigidity Hard to process
Polytetrafluoroethylene (PTFE), polyvinylidene fluoride (PVDF)	Commercial	MD	High mechanical strength and chemical resistance High thermal stability	Hydrophobicity Broader pore size distribution

#### 2.2.2. Inorganic Membranes

Inorganic membranes, encompassing ceramics, zeolites, and carbon-based materials, provide superior chemical and thermal stability, longer lifetime and higher antifouling property compared to their polymeric counterparts [48]. The most common inorganic membranes are glass membranes, ceramic membranes, metallic membranes, carbon membranes, and zeolitic membranes. Titania and silica membranes offer excellent hydrophilicity and resistance to oxidative degradation, while zeolite membranes provide well-defined microporous channels for molecular sieving [49,50,51]. However, high fabrication costs and brittleness restrict their widespread application. The application, advantages and disadvantages of different types of inorganic membranes are summarized in Table 2.

#### 2.2.3. Mixed Matrix Membranes

Mixed matrix membranes (MMMs) are a promising type of membranes in both gas separation and water process, where inorganic fillers are dispersed in a polymer matrix. The objective of MMMs is to provide an approach to overcome both the upper-bound of the trade-off between selectivity and permeability for polymeric membranes and the inherent brittleness and high cost of inorganic membranes. The inorganic fillers applied in MMMs can be either porous or nonporous. Commonly used inorganic fillers include zeolite, carbon molecular sieves, silica, metal oxide, carbon nanotubes, layered silicate, metal–organic frameworks and graphene [55].

### 2.3. Carbon Membranes

Carbon membranes (CMs), particularly carbon molecular sieve (CMS) membranes, have emerged as an intermediate solution combining the mechanical robustness of ceramics with the tunability of polymers. Based on the configurations, common carbon membranes can be categorized into two types: unsupported and supported. Unsupported carbon membranes are mainly flat sheets (film), capillary and hollow fibre, while the supported membranes are principally either flat sheets or tubes [56]. Carbon membranes consist of rigid amorphous carbon structures with slit-like ultramicropores (0.3~0.8 nm) that facilitate selective permeation of small molecules and vapor species [57]. The CMS structure is typically obtained by controlled carbonization of an organic polymer precursor under inert atmosphere, followed by stabilization and activation to develop accessible porosity [58]. Compared with polymeric membranes, CMS membranes can withstand high temperatures (>300 °C), aggressive solvents, and strong acids or bases. They have been widely investigated for gas separation (H_2_/CH_4_, O_2_/N_2_) [59,60,61] and are increasingly studied for water purification and desalination applications [62]. Their intrinsic hydrophobic-hydrophilic balance, combined with narrow pore-size distribution, enables near-complete salt rejection during pervaporation desalination.

Notable advances in carbon membranes include (i) hollow-fiber CMS modules for industrial-scale vapor separation [63], (ii) heteroatom doping (N, O, S) to tailor surface polarity and sorption selectivity [64], and (iii) integration of CMS with inorganic supports for enhanced mechanical integrity [65]. These developments demonstrate that precise control of pore architecture and surface functionality is key to achieving superior water transport and salt exclusion in carbon-based desalination membranes.

### 2.4. Formation Mechanism of Carbon Membranes

**Figure 3:**
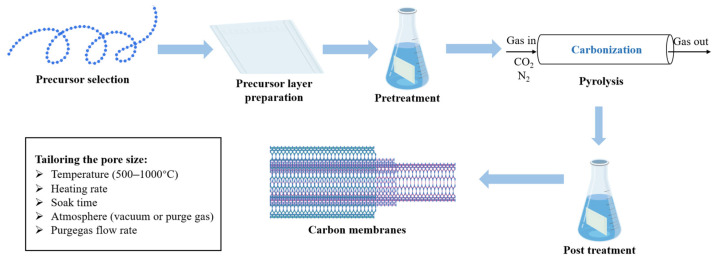
General processes for carbon membrane preparation.

The preparation of carbon membranes normally requires several steps and preparation method directly impacts the performance of the subsequent membrane. Generally, there are six processes in the preparation of a carbon membrane, as shown in Figure 3 [66]. During the whole preparation, many factors need to be considered for producing a high-performance carbon membrane.

The performance of a carbon membrane is primarily determined by its precursor chemistry and carbonization parameters. During pyrolysis, thermal decomposition of the precursor induces the elimination of heteroatoms (O, N, H) and the formation of sp^2^- and sp^3^-hybridized carbon frameworks [67]. The resulting pore structure depends strongly on the precursor’s molecular architecture, crosslinking density, and decomposition pathway [68]. For instance, polyimide and phenolic precursors generate dense, aromatic carbon networks with narrow ultramicropores, whereas polyacrylonitrile (PAN) yields graphitic domains with moderate mesoporosity [69,70,71].

Carbonization conditions, including temperature, atmosphere, dwell time, and heating rate, are vital factors affecting pore size and connectivity. Lower carbonization temperatures (400~600 °C) result in partially carbonized structures with limited conductivity and microporosity, while higher temperatures (>800 °C) enhance graphitization but may collapse ultramicropores [71]. Applicable carbonization atmosphere could be N_2_, Ar, CO_2_ or vacuum. The use of an inert gas enhances membrane permeability by improving heat and mass transfer, whereas vacuum carbonization promotes the formation of denser carbon structures characterized by smaller pore sizes and reduced d-spacings [71,72]. Hence, optimizing the carbonization conditions is essential to balance permeability and selectivity.

Another critical step is the preparation of the precursor film or coating, which influences the membrane morphology and defect formation. Spin-coating, dip-coating, and phase inversion are widely used methods for forming uniform polymer films on porous ceramic or metal supports [73,74,75]. The precursor concentration and solvent system govern the resulting layer thickness and surface smoothness.

Several studies reported green and low-temperature fabrication routes for CMS membranes, including hydrothermal pre-carbonization, templated pyrolysis using bio-derived additives (e.g., glucose, lignin), and chemical vapor deposition (CVD) of carbon nanolayers on supports [21,76]. Furthermore, machine-learning-assisted process control is emerging to predict optimal carbonization parameters for desired pore-size distributions of carbon materials [77,78].

## 3. Natural Polymer Precursors for Carbon Membranes

The growing demand for sustainable and environmentally friendly materials has motivated intensive research into renewable biomass resources as precursors for carbon membranes. Unlike petrochemical-based polymers, natural macromolecules such as cellulose, chitosan, lignin, and starch are abundant, biodegradable, and often derived from waste streams of agriculture or forestry industries. Critically, these natural polymers are consistently highlighted for their low cost and cost-effectiveness compared to synthetic alternatives, providing a compelling economic advantage for large-scale membrane production [79]. Their molecular architectures, composed mainly of polysaccharide, polyphenolic, or protein units, provide a rich variety of functional groups (-OH, -NH_2_, -COOH, -OCH_3_), which play crucial roles in the thermal degradation and carbonization pathways.

In contrast to synthetic polymers with uniform molecular structures, biopolymers exhibit hierarchical morphologies, crystalline-amorphous domains, and strong intermolecular hydrogen bonding [80]. These features influence carbonization behavior, leading to diverse pore structures and surface functionalities in the derived carbon materials. For instance, cellulose, due to its linear β-1,4-glucan backbone and high crystallinity, tends to yield well-ordered carbon sheets with narrow micropores, whereas lignin produces amorphous carbons rich in aromatic clusters and heteroatom dopants [81]. The inherent oxygen- and nitrogen-containing moieties in these biopolymers facilitate heteroatom doping during pyrolysis, improving surface polarity and hydrophilicity-properties advantageous for pervaporation desalination.

### 3.1. Cellulose and Its Derivatives

**Figure 4:**
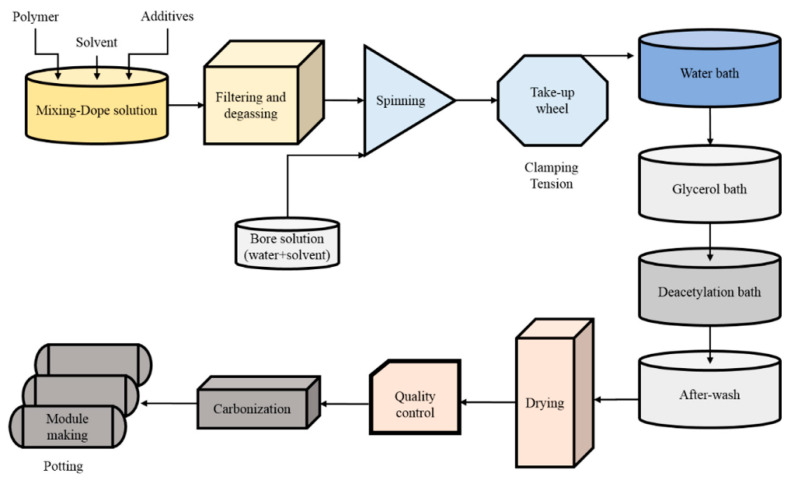
Schematic diagram of the pilot-scale fabrication process for carbon membranes from cellulose acetate hollow fibers [82].

Cellulose is the most abundant biopolymer and a benchmark precursor for biomass-derived carbon membranes. Its regular chain structure and extensive hydrogen bonding result in excellent mechanical strength, making it suitable for forming films or fibers prior to carbonization. Research on the production of carbon membranes using cellulose precursors has reached the pilot-scale stage [82], and its process flow is shown in Figure 4.

Previous studies have demonstrated the feasibility of cellulose-derived carbon membranes (CDCMs) for both gas and vapor separations. Araújo et al. fabricated cellulose-based CMS membranes via controlled pyrolysis of urea doped cellulose films, achieving a uniform carbon layer with bimodal pore size distribution (0.7~1 nm micropores and 0.35–0.70 nm ultramicropores) [17]. The resulting membranes exhibited high O_2_/N_2_ and H_2_/CH_4_ selectivity and stable gas permeation after 120 days aging. Meanwhile, incorporation with thermally labile additives such as polyvinylpyrrolidone (PVP) can create different porous structures, acting as a molecular spacer [83], while the addition of metallic fillers increased the micropore volume of the resulting carbon membranes [84]. Furthermore, chemical pre-treatment (e.g., acetylation or oxidation) can regulate the degree of polymerization and crystallinity, thereby controlling the carbon yield and pore connectivity [85].

### 3.2. Chitosan and Lignin

Chitosan, a deacetylated derivative of chitin, contains amino groups that act as intrinsic nitrogen dopants, facilitating the formation of N-doped carbon networks. Such doping improves the affinity toward water molecules and enhances selective adsorption of polar compounds [86,87]. When carbonized, chitosan can yield N-doped carbon membranes with high graphitization and good wettability, which was employed as protective layers on zinc anodes in aqueous Zn batteries [88].

Lignin, a byproduct of the paper industry, provides a cost-effective and aromatic-rich precursor. Due to its crosslinked phenylpropanoid structure, lignin produces carbons with high aromatic content and microporous/mesoporous features after carbonization at 700~900 °C [89]. Lignin-derived carbon membrane have recently gained attention for vapor separation and wastewater treatment due to their mechanical integrity and low precursor cost. For example, lignin derivative, lignocresol, has been used to prepare carbon membranes with submicron thickness and well-developed microporous structures which exhibited high selectivity for CO_2_/N_2_ and H_2_/CH_4_ [90].

### 3.3. Starch and Related Polysaccharides

Among natural polymers, starch offers distinct advantages such as low cost, abundance, and processability into various morphologies (films, gels, fibers). Structurally, starch consists of two polysaccharides, amylose and amylopectin, whose ratio and crystallinity strongly affect its carbonization behavior [91]. The application of raw starch outside of the food industry is limited, mostly because it has many undesirable properties like the insolubility in cold water and the tendency of retrogradation after physical treatment [92,93]. Therefore, to use starch as a biopolymer material, physical and/or chemical modifications are typically needed. Modifications can change the starch structure and affect the hydrogen bonding in a controllable way to improve and extend its applications. Common modifications of starch are cross-linking, stabilization, enzymatic hydrolysis, acid hydrolysis, derivatization, oxidation, dextrinization, lipophilic substitution, pre-gelatinization, and hydrothermal treatment [93]. While starch has long been employed as an additive or matrix material in polymeric membranes (e.g., starch/chitosan [94,95] or starch/PVA blends [96,97]), its potential as a sole carbon precursor has only recently gained recognition. It has been reported the fabrication of starch-derived carbon membranes exhibiting microporous structures and hydrophilic surfaces beneficial for vapor transport [98].

Other biomass-based materials such as sucrose and glucose have been used as small-molecule precursors to tune the pore morphology of CMS membranes. For example, sucrose-based carbon coatings applied onto alumina supports yielded continuous ultrathin films with high salt rejection (>99%) and improved hydrophilicity for pervaporation desalination [99]. In addition, glucose-derived carbon membranes prepared hydrothermally at 200 °C for 5 h formed uniform amorphous carbon layers with ~0.3 nm pores, exhibiting high gas selectivity [100].

### 3.4. Carbonization Mechanisms and Structural Control

Despite the diversity of biopolymer sources, their thermal decomposition follows a general pattern: dehydration, depolymerization, and aromatization [101,102]. The decomposition onset typically occurs between 250~350 °C, accompanied by the release of CO_2_, CO, and H_2_O [102]. Crosslinking reactions between fragmented intermediates lead to the formation of polyaromatic clusters, which gradually coalesce into a continuous carbon matrix at higher temperatures [103].

To tailor pore architecture, activation techniques (physical or chemical) are frequently applied. Physical activation with CO_2_ or steam enlarges micropores, whereas chemical activation using KOH, ZnCl_2_, or phosphoric acid generates mesoporous frameworks [103,104,105,106]. However, excessive activation may compromise structural integrity and reduce salt rejection in desalination membranes.

In summary, natural polymer precursors have emerged as sustainable alternatives for fabricating carbon membranes, bridging the gap between high-performance inorganic materials and environmentally responsible production. Cellulose, chitosan, lignin, starch, and other biopolymers each offer unique chemical and structural attributes that influence carbon yield, pore morphology, and surface chemistry. Nevertheless, achieving uniform films, scalable fabrication, and consistent pore structures remain the main challenges for future exploration.

## 4. Pervaporation Desalination Using Carbon Membranes

### 4.1. Principles and Advantages of Pervaporation Desalination

Pervaporation (PV) is particularly suited for carbon membrane applications in desalination for two key reasons: its ability to handle hypersaline or chemically aggressive feeds that challenge pressure-driven processes like RO, and its operation with a dense selective layer which minimizes fouling and pore wetting issues common in MD.

**Figure 5:**
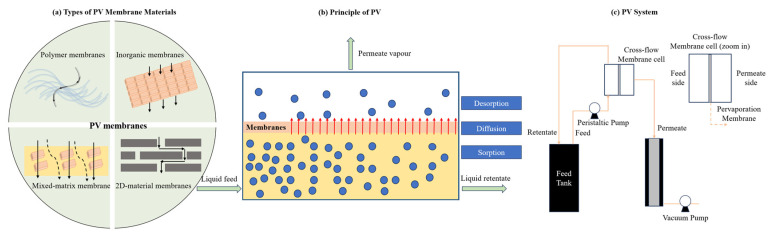
Schematic diagram of the pervaporation process: (**a**) key membrane material, (**b**) solution-diffusion separation mechanism, and (**c**) process unit.

PV is a thermally driven membrane process in which a liquid feed is partially vaporized through a nonporous or ultramicroporous membrane, as illustrated by Figure 5 [35]. The separation is governed by a solution-diffusion mechanism: (i) selective sorption of the permeant at the feed-membrane interface, (ii) diffusion across the membrane matrix, and (iii) desorption and condensation on the permeate side under vacuum or sweep gas [107]. In desalination applications, water preferentially permeates the membrane, while salts and nonvolatile solutes are retained in the feed phase.

Compared with RO and MD, PV operates at moderate temperatures (50~80 °C) and does not require high hydrostatic pressure, which makes PV particularly advantageous for treating hypersaline brines and industrial wastewater concentrates [108]. Furthermore, PV membranes can maintain high salt rejection (>99.9%) because salt ions, being nonvolatile, cannot pass through the dense selective layer. The simplicity of PV system configuration also enables hybrid integration with solar or waste-heat sources for low-carbon desalination [109]. The successful application of carbon membranes embodying these principles is detailed in the following section, with case studies highlighting their performance and the involved chemistry.

### 4.2. Carbon Membranes for Pervaporation Desalination

Carbon membranes have gained increasing attention for PV desalination owing to their thermal robustness, chemical resistance, and ultramicroporous structures that permit selective water vapor transport, providing an alternative to polymeric and ceramic membranes that often suffer from swelling or hydrolytic degradation under high salinity and temperature. The hydrophobic graphitic backbone combined with polar surface functionalities creates an optimal balance for water adsorption and diffusion while rejecting ions and organics. Recent developments have focused on tailoring carbon microstructures, pore connectivity, and surface chemistry by selecting appropriate organic precursors and controlling carbonization parameters to achieve high water flux and excellent salt rejection.

The preparation of starch-derived carbon membranes on porous alumina supports, which demonstrated that natural polysaccharides can serve as effective carbon sources for PV desalination [98]. Modified starches such as gelatinized, acid-hydrolyzed, and mixed-modified starch enabled the formation of stable coating suspensions and continuous carbon layers after dip-coating and carbonization. At a carbonization temperature of about 700 °C, the membranes exhibited a pure amorphous carbon structure and achieved a water flux of 4.98 L/m^2^/h with 92.6% salt rejection at 1 wt% NaCl and 70 °C [98]. Following similar bio-derived routes, sucrose- and sorbitol-based carbon membranes were reported as low-cost, renewable alternatives. Sucrose-derived membranes carbonized at 300 °C on alumina tubes yielded water fluxes between 7~23 L/m^2^/h and salt rejection above 97%, maintaining nearly 100% rejection for 60 h of continuous operation at 60 °C [99]. Sorbitol-derived membranes, produced under N_2_ at 350 °C, achieved outstanding performance: 17.35 kg/m^2^/h water flux and >99.9% salt rejection at 3.5 wt% NaCl and 60 °C, remaining stable for 100 h, highlighting the potential of sugar alcohols as efficient carbon precursors for long-term PV desalination [110].

In addition to natural polymers, synthetic resins have also been utilized. Phenolic-resin-derived carbon membranes fabricated through a two-step wet/dry coating on freeze-cast alumina substrates showed a well-developed micro/mesoporous carbon layer. When carbonized at 800 °C, these membranes delivered water fluxes up to 14.6 L/m^2^/h with 99% salt rejection for 1~5 wt% NaCl feeds at 25~75 °C [75]. The two-step phase inversion ensured defect-free coating and superior mechanical adhesion compared with conventional single-step methods.

Efforts have also been directed toward hybrid and composite carbon membranes to enhance hydrophilicity and flux. A notable example is the carbon-silica hybrid membrane prepared via sol–gel condensation of TEOS/TEVS and P123 surfactant, followed by vacuum carbonization at 450 °C. The resulting interlayer-free hybrid membrane achieved 26.5 L/m^2^/h water flux with 99.5% rejection at 1 wt% NaCl and 60 °C, outperforming many polymeric PV membranes due to the synergistic microporous network [111]. Similarly, PVA/carbon nanotube composite membranes attained 6.96 kg/m^2^/h flux and 99.91% rejection at 22 °C, benefitting from hydrogen-bond-mediated water transport through nanoscale carbon pathways [112]. The desalination performance of representative carbon membranes and comparative PV membranes is summarized in Table 3, highlighting their material composition, operating conditions, and performance metrics.

Generally, the performance of carbon-based PV membranes depends on a delicate interplay among pore structure, surface chemistry, and operation conditions. Ultramicropores are essential for water vapor diffusion and salt rejection, while excessive mesoporosity can lead to nonselective permeation and decreased rejection. Moderate hydrophilicity enhances water sorption without promoting capillary condensation. Higher feed temperature and vacuum level accelerate water vaporization but may cause membrane stress. Additionally, fouling and wetting remain critical challenges for industrial deployment.

Beyond experimental studies, molecular dynamics simulations have provided mechanistic insights into water transport within carbon frameworks. For instance, a carbon honeycomb (CHC) membrane model exhibited theoretical fluxes exceeding 3.9 × 10^4^ L/m^2^/h with complete salt rejection at 320 K, revealing the fundamental influence of pore diameter (6~13 Å) and membrane thickness on selective vapor transport [117].

In summary, carbon membranes for PV desalination demonstrate remarkable versatility in precursor selection, from renewable carbohydrates to engineered hybrid networks, while maintaining high salt rejection and tunable permeability. Natural polymer-derived carbon membranes are emerging as sustainable candidates due to their low cost, abundance, and ease of carbonization at moderate temperatures. Continued progress in controlling pore architecture, surface polarity, and defect density will be pivotal in translating these laboratory-scale findings into scalable, high-performance membranes for sustainable desalination.

## 5. Challenges and Future Perspectives

### 5.1. Scalability and Manufacturing Challenges

Although significant progress has been made in developing high-performance carbon and hybrid membranes for desalination, large-scale commercialization remains limited. One major challenge lies in fabrication scalability. Conventional carbon membrane production involves high-temperature carbonization (600~900 °C) in inert atmospheres, which demands high energy input and precise control of heating rates, leading to increased manufacturing cost. Moreover, the intrinsic brittleness of carbon layers makes them susceptible to cracking during pyrolysis or module assembly, reducing yield and reproducibility.

To address these limitations, emerging low-temperature carbonization routes like hydrothermal carbonization have been proposed [118]. Similarly, microwave-assisted carbonization allows rapid and uniform heating, shortening fabrication time while preserving pore structure [119]. These innovations point toward more energy-efficient and scalable manufacturing methods that align with low-carbon production principles.

### 5.2. Structural Stability and Long-Term Durability

For carbon membranes derived from natural polymer precursors, maintaining structural integrity during long-term pervaporation operation remains challenging. The thin carbon layer obtained from bio-based precursors often exhibits residual stress and moderate mechanical strength, making it vulnerable to cracking, delamination, or pore coarsening under cyclic thermal and hydraulic conditions. Moreover, prolonged exposure to saline or oxidizing environments can gradually alter surface functionality, thereby affecting hydrophilicity and selectivity.

Enhancing interfacial adhesion through graded carbon–ceramic transitions, optimizing carbonization to reduce internal stress, and incorporating flexible or graphitized structures are promising strategies to improve stability. Future work should emphasize durability evaluation under continuous operation to ensure that bio-derived carbon membranes sustain their high selectivity and flux over extended service lifetimes.

### 5.3. Data-Driven Design and Digitalization

The optimization of carbon membranes has traditionally relied on empirical trial-and-error methods, which are time-consuming and material-intensive. In recent years, data-driven modeling and artificial intelligence (AI) have emerged as transformative tools for accelerating membrane design [120,121,122]. Machine learning algorithms can correlate precursor chemistry, carbonization parameters, and pore-structure descriptors with experimental performance data, enabling predictive modeling of flux and selectivity. Combined with high-throughput experiments and automated synthesis, such digital workflows could dramatically shorten development cycles. The concept of a “digital twin” membrane reactor, where real-time process data feed into predictive control algorithms, is also under exploration for continuous carbonization and defect monitoring.

## 6. Conclusions

Carbon membranes derived from natural polymers offer a sustainable and high-performance solution for desalination. This review demonstrates their exceptional potential in pervaporation, with specific precursors achieving benchmark results: sorbitol-derived membranes attain fluxes up to 17.35 kg/m^2^/h with >99.9% salt rejection, while sucrose-derived membranes provide 7–23 L/m^2^/h with >97% rejection. The critical link between precursor chemistry (e.g., inherent O/N functional groups) and carbonization conditions (optimized at 300–700 °C) enables the formation of ultra-micropores essential for selective water vapor transport.

Despite this promise, scalability and durability remain primary challenges. The brittleness of carbon layers and the energy intensity of high-temperature pyrolysis hinder large-scale adoption. Future progress depends on developing low-energy carbonization routes (e.g., microwave or hydrothermal), enhancing mechanical integrity through composite structures, and employing data-driven design to optimize membrane microstructure.

In summary, natural polymer-derived carbon membranes merge high selectivity (>99% rejection), competitive flux, and robust stability. Addressing the current manufacturing and mechanical challenges will position these sustainable materials as pivotal components in the next generation of energy-efficient desalination technologies.

## Figures and Tables

**Table 2:** Comparison of different inorganic membranes [48,52,53,54].

Membrane Types	Technology Maturity	Applications	Advantages	Disadvantages
Metallic membranes (mostly Pd-based membranes)	Under development	Separation of hydrogen	Mechanical strength High permeating flux Thermal stability	Very high cost Surface poisoning
Ceramic membranes	Commercial	UF MF Gas separation Membrane reactor	Chemical, thermal and mechanical stability No swelling in any solvent Resistant to chemicals	High costs and complex fabrication compared to polymeric membranes Lower packing densities than polymeric membranes
Glass membranes	Commercial	Gas separation Membrane reactor Pervaporation	High selectivity Chemical and thermal stability Simple fabrication Cheaper	Degraded by water
Carbon membranes	Under development	Gas separation	Molecular sieving High efficiency	Very fragile Degraded by water and some organic compounds
Zeolitic membranes	Under development	RO Gas separation Pervaporation	Uniform and very narrow pore size Catalytic properties	High material cost Poor process ability Negative thermal expansion

**Table 3:** Desalination performance of carbon membranes and other PV membranes.

Membrane Type	Feed	Temp. (°C)	Water Flux (L/m^2^/h)	Salt Rejection (%)	Ref.
Starch-derived carbon membrane	1 wt% NaCl	70	4.98	92.6	[98]
Sucrose-derived carbon membrane	1~7 wt% NaCl	60	7~23	≥97	[99]
Sorbitol-derived carbon membrane	3.5 wt% NaCl	60	17.35	>99.9	[110]
Phenolic resin-derived carbon membrane	1–5 wt% NaCl	75	14.6	99	[75]
Carbon–silica hybrid membrane	1 wt% NaCl	60	26.5	99.5	[111]
PVA/CNT composite membrane	3.5 wt% NaCl	22	6.96	99.91	[112]
Sulfonic acid functionalized PVA/PAN composite membrane	3.5 wt% NaCl	70	46.3	99.8	[113]
ZSM-5 thin membrane	3 wt% NaCl	80	10.4	99.5	[114]
TEOS-MTES silica membrane	1 wt% NaCl	30	2	98	[115]
Ultrathin MXene membrane	3.5 wt% NaCl	60	85.4	99.5	[116]

## Data Availability

No new data were created or analyzed in this study. Data sharing is not applicable to this article.
